# Regulation of the pentose phosphate pathway by an androgen receptor–mTOR-mediated mechanism and its role in prostate cancer cell growth

**DOI:** 10.1038/oncsis.2014.18

**Published:** 2014-05-26

**Authors:** E Tsouko, A S Khan, M A White, J J Han, Y Shi, F A Merchant, M A Sharpe, L Xin, D E Frigo

**Affiliations:** 1 Center for Nuclear Receptors and Cell Signaling, Department of Biology and Biochemistry, University of Houston, Houston, TX, USA; 2 Department of Engineering Technology, University of Houston, Houston, TX, USA; 3 Department of Neurosurgery, Houston Methodist Research Institute, Houston, TX, USA; 4 Department of Molecular and Cellular Biology, Baylor College of Medicine, Houston, TX, USA; 5 Department of Pathology and Immunology, Baylor College of Medicine, Houston, TX, USA; 6 Dan L. Duncan Cancer Center, Houston, TX, USA; 7 Center for Genomic Medicine, Houston Methodist Research Institute, Houston, TX, USA

**Keywords:** prostate cancer, G6PD, pentose phosphate pathway, androgen receptor, mTOR, PTEN

## Abstract

Cancer cells display an increased demand for glucose. Therefore, identifying the specific aspects of glucose metabolism that are involved in the pathogenesis of cancer may uncover novel therapeutic nodes. Recently, there has been a renewed interest in the role of the pentose phosphate pathway in cancer. This metabolic pathway is advantageous for rapidly growing cells because it provides nucleotide precursors and helps regenerate the reducing agent NADPH, which can contribute to reactive oxygen species (ROS) scavenging. Correspondingly, clinical data suggest glucose-6-phosphate dehydrogenase (G6PD), the rate-limiting enzyme of the pentose phosphate pathway, is upregulated in prostate cancer. We hypothesized that androgen receptor (AR) signaling, which plays an essential role in the disease, mediated prostate cancer cell growth in part by increasing flux through the pentose phosphate pathway. Here, we determined that G6PD, NADPH and ribose synthesis were all increased by AR signaling. Further, this process was necessary to modulate ROS levels. Pharmacological or molecular inhibition of G6PD abolished these effects and blocked androgen-mediated cell growth. Mechanistically, regulation of G6PD via AR in both hormone-sensitive and castration-resistant models of prostate cancer was abolished following rapamycin treatment, indicating that AR increased flux through the pentose phosphate pathway by the mammalian target of rapamycin (mTOR)-mediated upregulation of G6PD. Accordingly, in two separate mouse models of Pten deletion/elevated mTOR signaling, Pb-Cre;Pten^f/f^ and K8-CreER^T2^;Pten^f/f^, G6PD levels correlated with prostate cancer progression *in vivo*. Importantly, G6PD levels remained high during progression to castration-resistant prostate cancer. Taken together, our data suggest that AR signaling can promote prostate cancer through the upregulation of G6PD and therefore, the flux of sugars through the pentose phosphate pathway. Hence, these findings support a vital role for other metabolic pathways (that is, not glycolysis) in prostate cancer cell growth and maintenance.

## Introduction

Prostate cancer is a heterogeneous and complex disease that is the second leading cause of cancer-related death in men. During the cancer's pathogenesis, androgen receptor (AR) signaling plays a central role by supporting aberrant cell growth.^1^ As such, hormone ablation is the standard of care for advanced prostate cancer. Unfortunately, current AR-targeted therapies do not stop disease recurrence. Interestingly, castration-resistant prostate cancers (CRPCs) still often express functional AR and rely on its activity for continued growth. Hence, understanding the downstream processes that modulate AR's growth effects may yield new therapeutic approaches to help manage the disease.

Since the description of the ‘Warburg effect' in the 1920s, it has been known that cancer cells can metabolize glucose via aerobic glycolysis and thus, have an increased demand for sugars.^2^ This observation kept scientific focus on glycolysis and overshadowed other aspects of glucose metabolism that could also promote cancer by providing the precursors and energy required for a rapidly growing cell. Hence, in addition to glycolysis, alternative glucose metabolic pathways may also promote a malignant phenotype.^3, 4^ Correspondingly, recent studies support an oncogenic role for non-glycolytic metabolic pathways and demonstrate that the shuttling of metabolites through specific metabolic pathways can rapidly shift to accommodate tumor requirements.^4, 5, 6^

The activity of phosphofructokinase-1 (PFK1), the rate-limiting enzyme of glycolysis, is regulated by phosphofructokinase-2/fructose-2,6-bisphosphatase (PFK2) isoenzymes. These enzymes display a tissue-specific expression and activity. Interestingly, 6-phosphofructo-2-kinase/fructose-2,6-biphosphatase 4 (PFKFB4), an isoform of PFK2, was required for the survival of prostate cancer cells but not benign prostate epithelial cells.^7^ This is significant because this isoform has a greater phosphatase activity relative to its kinase activity and therefore favors the breakdown of fructose 2,6-bisphosphate, an allosteric activator of PFK1. This led to an accumulation of fructose-6-phosphate and its subsequent shifting away from glycolysis. Additionally, the glycosylation/inhibition of PFK1 promoted cancer cell growth *in vitro* and tumor formation *in vivo*.^8^ Finally, the glycolytic enzyme pyruvate kinase M2 is expressed in most human tumors.^9, 10, 11, 12^ This enzyme typically exists in its less active, dimeric form in cancers due to the actions of various oncoproteins as well as post-translational modifications.^13, 14, 15, 16^ Collectively, all of these diverse actions function to block the flux through glycolysis. As a result, upstream metabolic intermediates begin to accumulate and can be rerouted into the pentose phosphate pathway for tumorigenic processes.

The pentose phosphate/hexose monophosphate shunt pathway is an alternative metabolic pathway for glucose breakdown. Upon transportation of glucose into the cell via glucose transporters, the enzyme hexokinase converts glucose to glucose-6-phosphate. Glucose-6-phosphate can then be metabolized further via glycolysis or the pentose phosphate pathway.^17^ The pentose phosphate pathway synthesizes precursors for nucleotide biosynthesis and generates NADPH.^18^ Depending on the cell requirements, NADPH can be utilized for anabolic reactions as well as maintaining cellular redox homeostasis. Because of the large biosynthetic demands of a rapidly growing cancer and their need to adapt to stressful environments, the pentose phosphate pathway has been suggested to promote cancer progression and therapy resistance.^19^ Accordingly, many of the enzymes that make up the pentose phosphate pathway are associated with malignancy.^20^ Additional data has more recently demonstrated a functional role for this pathway in aggressive prostate cancer.^7^

The pentose phosphate pathway is comprised of two phases: the oxidative and non-oxidative phases. The rate-limiting enzyme of the pentose phosphate pathway is glucose-6-phosphate dehydrogenase (G6PD). G6PD is responsible for the oxidation of glucose-6-phosphate to 6-phosphoglucono-δ-lactone and generates NADPH as a byproduct.^17, 18^ G6PD has been found to be overexpressed in multiple cancers,^21, 22, 23^ and is negatively regulated by the tumor suppressor p53.^24^ In prostate cancer, G6PD was suggested as a biomarker for prostatic carcinoma more than 30 years ago.^25^ Consequently, G6PD activity was found to be four times higher in prostate cancer compared with benign prostatic hyperplasia.^25, 26^ These data are supported by recent studies that indicate that both G6PD levels and metabolism through the pentose phosphate pathway are increased in prostate cancer.^7, 27, 28^ Here, we investigated whether AR signaling regulated the pentose phosphate pathway and what role, if any, this played in prostate cancer progression. We surmized that, given the requirement of tumor cells for biosynthetic precursors and the reducing power needed both for anabolic reactions and to combat oxidative stress, AR may shift cellular metabolism to accommodate such demands.

## Results

### G6PD is required for prostate cancer cell proliferation

In 1982, Zampella *et al.*^25^ suggested that G6PD could serve as a clinical indicator for prostate cancer, where G6PD activity was four times higher in carcinomas than in benign prostatic hyperplasia. Soon after this initial study, an independent group demonstrated that G6PD activity correlated with Gleason score.^26^ Subsequent studies have confirmed G6PD overexpression in different types of cancer.^21, 22, 23, 29^ Accordingly, silencing of G6PD has been reported to induce apoptosis,^30^ and increase ROS levels,^31^ while overexpression has been linked to enhanced tumor growth.^32^

**Figure 1:**
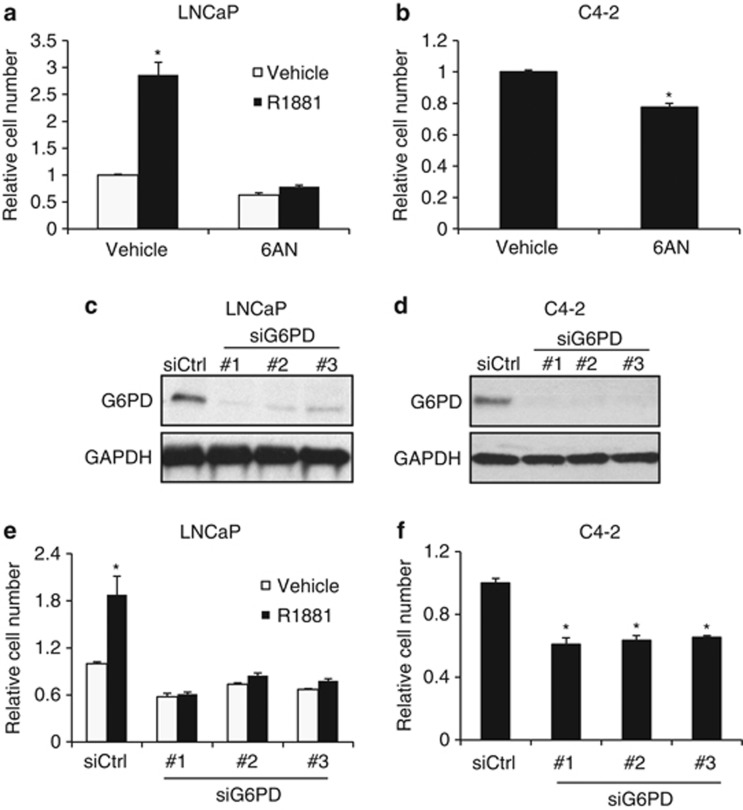
Inhibition of G6PD suppresses prostate cancer cell proliferation. (**a**) LNCaP cells were treated ± 100 nM 6-aminonicotinamide (6AN) ± the synthetic androgen R1881 for 7 days. Relative cell numbers were quantified after cell lysis by a fluorescent DNA-binding dye. (**b**) C4-2 cells, a CRPC-derivative of LNCaP cells, were treated ± 100 nM 6AN for 7 days. Relative cell numbers were quantified as in **a**. (**c** and **d**) LNCaP (**c**) and C4-2 (**d**) cells were transfected with siRNAs targeting scramble control (siCtrl) or G6PD (nos 1–3). After 72 h transfection, cells were harvested and subjected to immunoblot analysis using GAPDH as a loading control. (**e** and **f**) LNCaP (**e**) and C4-2 (**f**) cells were transfected with siRNAs as described in **c** and **d**, and then treated ± R1881 as indicated for 7 days. Relative cell numbers were quantified as in **a**. Representative results are expressed as mean relative cell number ±s.e. *, significant (*P*<0.05) changes from vehicle (no R1881; **a** and **e**) or control (**b** and **f**).

In prostate cancer, the regulation and functional role of G6PD is unclear. We hypothesized that G6PD could play a role in prostate cancer cell proliferation and further hypothesized that this could be regulated by AR, a central factor in prostate cancer. To test our hypothesis, we first blocked G6PD activity using a competitive inhibitor of G6PD, 6-aminonicotinamide (6AN), using a previously defined inhibitory concentration^33^ that we confirmed with preliminary dose–response experiments (data not shown). We then treated the human AR+, hormone-sensitive prostate cancer cell lines LNCaP (Figure 1a) and LAPC4 (Supplementary Figure 1A), as well as the CRPC-derivative C4-2 (Figure 1b) and 22Rv1 (Supplementary Figure 1B) cell models with 6AN and observed that this drug caused a significant decrease in cell growth. Further, androgen-mediated cell growth was completely blocked. Owing to the potential off-target effects of pharmacological inhibitors, we complemented our 6AN studies using small interfering RNAs (siRNAs) targeting G6PD. We confirmed silencing of G6PD by immunobloting in LNCaP, C4-2, CWR22 and 22Rv1 cells and demonstrated that, like 6AN, molecular inhibition of G6PD decreased prostate cancer cell growth in the presence or absence of androgens in both hormone-sensitive and castration-resistant cell models (Figures 1c–f; Supplementary Figures 1C–F). These results indicate that maximal prostate cancer cell growth, and particularly AR-driven cell growth, is G6PD dependent.

### G6PD inhibition lowers NADPH levels and controls the cellular redox state

**Figure 2:**
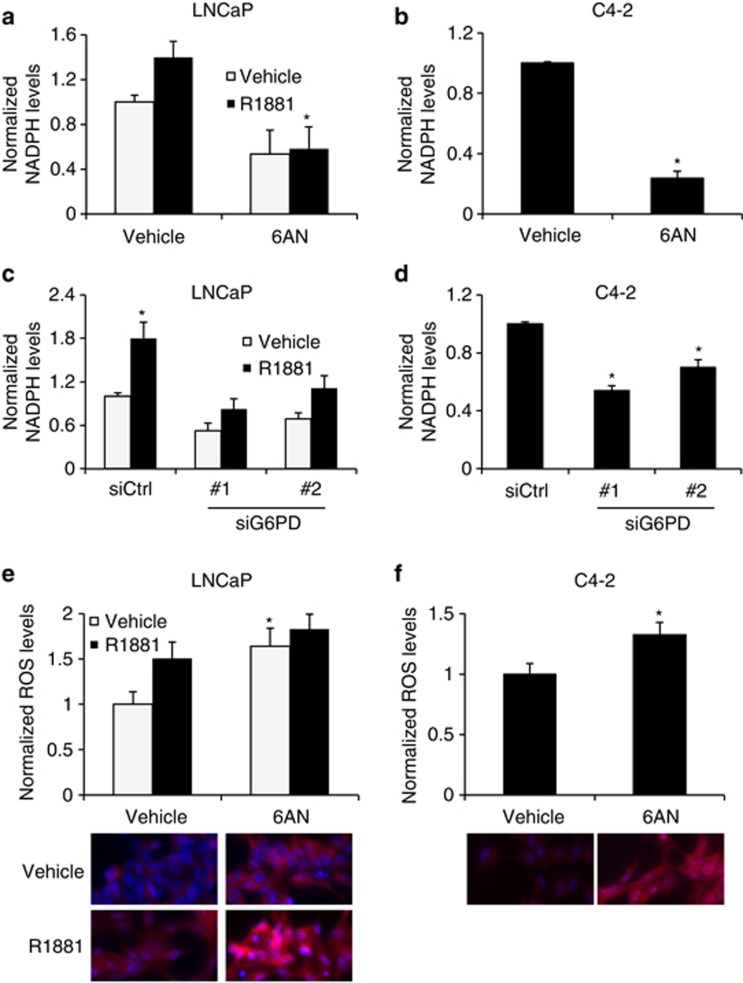
G6PD is required for maintaining NADPH levels and redox homeostasis. (**a** and **b**) LNCaP (**a**) or CRPC C4-2 (**b**) cells were treated ± 100 nM 6AN ± 10 nM R1881 as indicated for 3 days. Cells were lysed and NADPH was measured using an enzyme-recycling reaction and subsequent fluorescence measurement and normalized to total protein concentration as described in the Materials and methods. *, significant (*P*<0.05) changes from vehicle (no 6AN). (**c** and **d**) Using siRNA targeting scramble control (siCtrl) or the two most efficacious siRNAs targeting G6PD (nos 1 and 2), as determined in Figure 1, LNCaP (**c**) and C4-2 (**d**) cells were transfected with indicated siRNAs alone (**d**) or then treated for 3 days ± R1881 (**c**). NADPH levels were then measured as described in **a** and **b**. *, significant (*P*<0.05) changes from vehicle (no R1881; **c** or siCtrl, **d**). (**e** and **f**) To quantitate intracellular ROS levels, LNCaP (**e**) and C4-2 (**f**) cells were treated as described in **a** and **b**, respectively. After 3 days treatment, cells (∼300–1000 cells per group) were co-stained with Hoechst (blue) and MitoSOX Red (detects mitochondria-derived ROS) and fixed overnight. Cells were then imaged at × 20 the next day and images were analyzed using ImageJ software. Graphs show relative ROS levels per cell. *, significant (*P*<0.05) changes from vehicle (no 6AN). Below the graphs are representative images of the ROS staining.

One of the major products of the pentose phosphate pathway is NADPH. NADPH is a reducing agent that can be used for biosynthetic reactions and cellular detoxification. If AR signaling is indeed using the pentose phosphate pathway, AR signaling should correlate with NADPH levels, an effect that would be dependent on G6PD. We thus measured NADPH levels in LNCaPs following androgen treatment. Androgens increased NAPDH levels in a dose-dependent manner (Supplementary Figure 2). Next, we examined whether G6PD inhibition by 6AN treatment or silencing of G6PD caused a decrease in NADPH levels. Both hormone-sensitive LNCaP cells treated with androgens (Figures 2a and c) and CRPC C4-2 (Figures 2b and d) cells required active G6PD for NADPH production.

NADPH generated via the pentose phosphate pathway is a coenzyme for glutathione reductase needed to convert oxidized glutathione (GSSG) to its reduced form (GSH), which is used to regulate ROS levels. We investigated whether inhibition of G6PD altered ROS levels using a quantitative microscopy assay. Similar to what we^34^ and others^35, 36, 37^ have shown, androgens increased intracellular ROS levels in LNCaP cells (Figure 2e), likely as a result of increased mitochondrial metabolism,^38^ and hence, ROS byproducts. 6AN treatment increased both basal- and R1881-mediated ROS levels, suggesting 6AN was blocking the cells' antioxidant defense (Figure 2e). 6AN also increased ROS levels in C4-2 cells (Figure 2f). Although there is a delicate threshold where moderate levels of ROS activate signaling pathways to promote cell growth, higher ROS levels may stimulate apoptosis. Therefore, impaired G6PD activity could lead to cytotoxic levels of ROS. Taken together, these results highlight the regulation of NAPDH and ROS levels by androgens and the importance of G6PD in maintaining prostate cancer cell redox homeostasis.

### G6PD is required for nucleic acid synthesis

**Figure 3:**
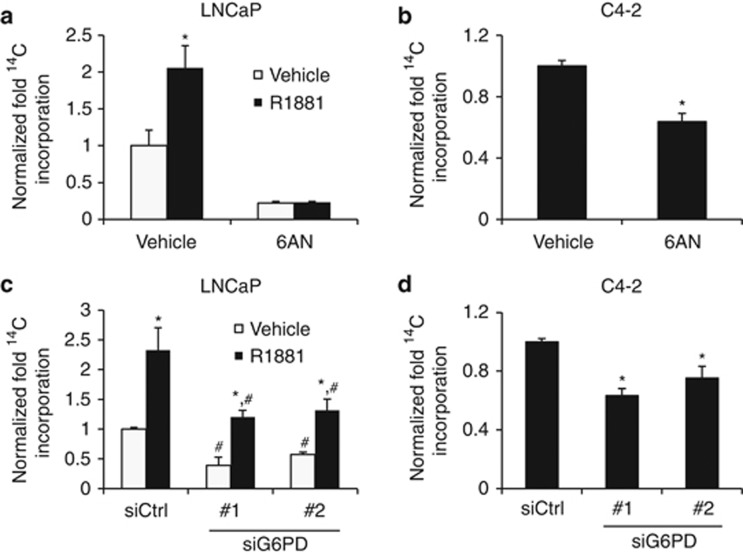
G6PD regulates ribose synthesis in prostate cancer cell models. (**a** and **b**) LNCaP (**a**) and C4-2 (**b**) cells were treated for 3 days as indicated ± 100 nM 6AN ± 10 nM R1881 prior to starvation and treatment with ^14^C-labeled glucose for an additional 24 h. Total RNA extraction was performed and the fraction of newly radiolabeled RNA was quantitated via a scintillation counter and normalized to the total RNA pool. *, significant (*P*<0.05) changes from vehicle (no R1881; **a**) or vehicle (no 6AN; **b**). (**c** and **d**) LNCaP (**c**) and C4-2 (**d**) cells were initially transfected and treated as in Figures 2c and d. Cells were then treated for an additional 24 h with ^14^C-labeled glucose and subjected to the ^14^C RNA incorporation assay as described above. *, significant (*P*<0.05) changes from vehicle (no R1881; **c**). ^#^, significant (*P*<0.05) changes from control (siCtrl; **c**). *, significant (*P*<0.05) changes from control (siCtrl; **d**).

In addition to NADPH, one of the major functions of the pentose phosphate pathway is to generate precursors for nucleotide synthesis. Providing these building blocks would be especially important for rapidly dividing cancer cells. Hence, we evaluated flux through the pentose phosphate pathway by tracking the incorporation of ^14^C from radiolabeled glucose into newly synthesized RNA and compared that to the total RNA pool. Both the basal- and androgen-mediated flux through the pentose phosphate pathway were inhibited by pharmacological (Figures 3a and b) or molecular (Figures 3c and d) inhibition of G6PD. This suggests that G6PD supports cancer cell growth in part by regulating the generation of nucleic acid precursors.

### AR signaling increases G6PD levels in hormone-sensitive and CRPC cell models

**Figure 4:**
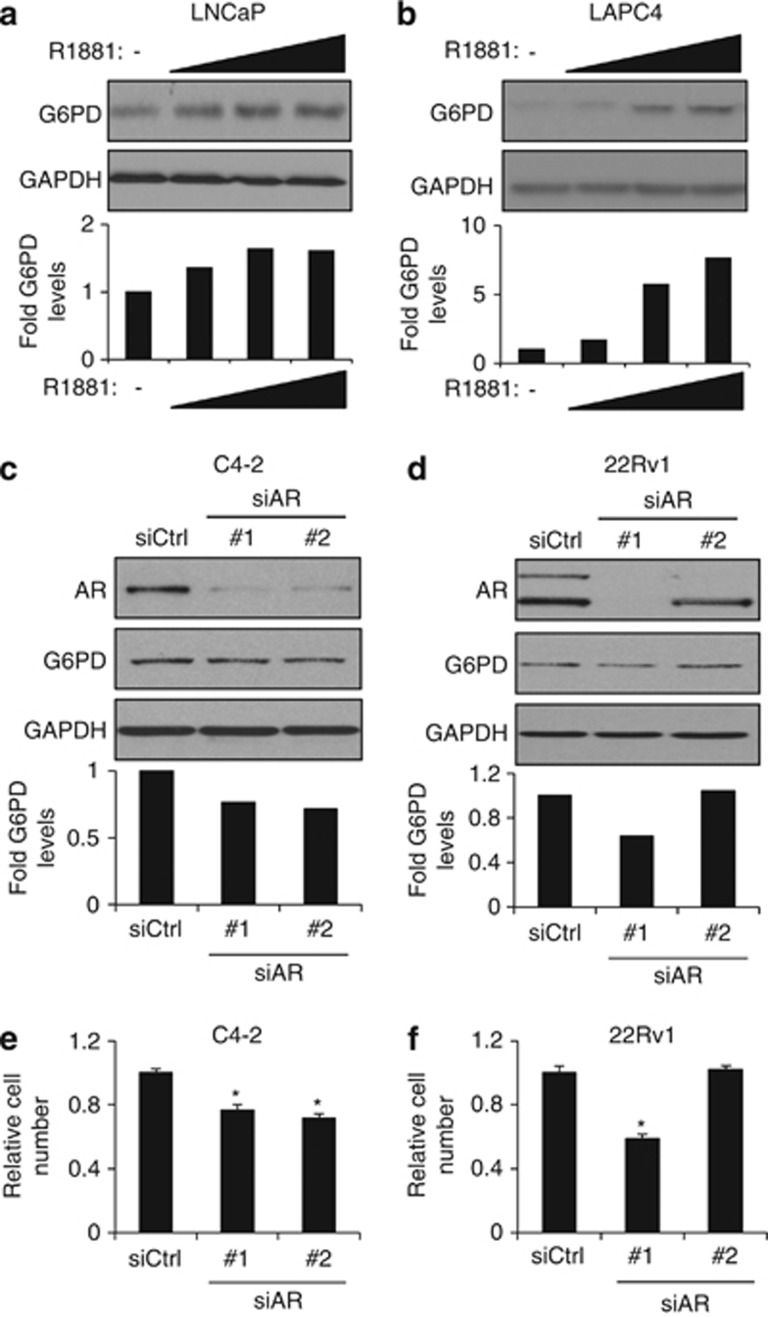
AR signaling regulates G6PD levels. (**a** and **b**) Hormone-sensitive LNCaP (**a**) or LAPC4 (**b**) cells were treated with increasing concentrations of R1881 (0, 0.1, 1 and 10 nM) for 72 h. Cells were harvested and subjected to immunoblot analysis using GAPDH as a loading control. (**c**–**f**) CRPC C4-2 (**c** and **e**) and 22Rv1 (**d** and **f**) cells were transfected with siRNAs targeting control (siCtrl) or AR (nos 1 and 2). Note, siAR #1 targets both full-length AR and the constitutive active, C-terminal-truncated AR splice variants found in 22Rv1 cells while siAR #2 targets only full-length AR. (**c** and **d**) After 72 h transfection, cells were lysed and subjected to immunoblot analysis using GAPDH as a loading control. Representative images (top) and graphs representing relative band density normalized to loading control (bottom) are shown for each subfigure. (**e** and **f**) Cell growth was then quantitated after 7 days as described in Figure 1. *, significant (*P*<0.05) changes from control (siCtrl).

Given that androgens increased flux through the pentose phosphate pathway, an effect that was dependent on G6PD, we suspected that AR signaling may regulate the levels of this rate-limiting enzyme. In LNCaP (Figure 4a), LAPC4 (Figure 4b) and VCaP (Supplementary Figure 3A) hormone-sensitive cell lines, G6PD protein levels were increased following androgen treatment. Interestingly, comparison of hormone-sensitive prostate cancer cell models to their CRPC-derivative models often demonstrated higher basal G6PD levels in the CRPC models, perhaps reflecting the high basal levels of AR activity in these cells (Supplementary Figure 3B). Knockdown of AR in CRPC cell models using two different siRNAs, one that silenced both full-length and C-terminally truncated, constitutively active splice variants (siAR #1) and one that targeted only full-length AR (siAR #2), decreased G6PD protein levels (Figures 4c and d) in a manner that correlated with AR-driven CRPC cell growth (Figures 4e and f; note, 22Rv1 cell growth is driven by the constitutive active splice variants of AR).^39^ To determine whether the AR regulation was at the transcriptional level, we measured G6PD mRNA levels in LNCaP, LAPC4 and VCaP cells (Supplementary Figure 3C) and observed moderate increases in G6PD mRNA following androgen treatment in a manner that did not consistently track with protein levels. Hence, while some regulation occurred at the transcriptional level, it appeared there is also post-transcriptional regulation.

### AR regulates G6PD levels and thus the pentose phosphate pathway through mTOR signaling

**Figure 5:**
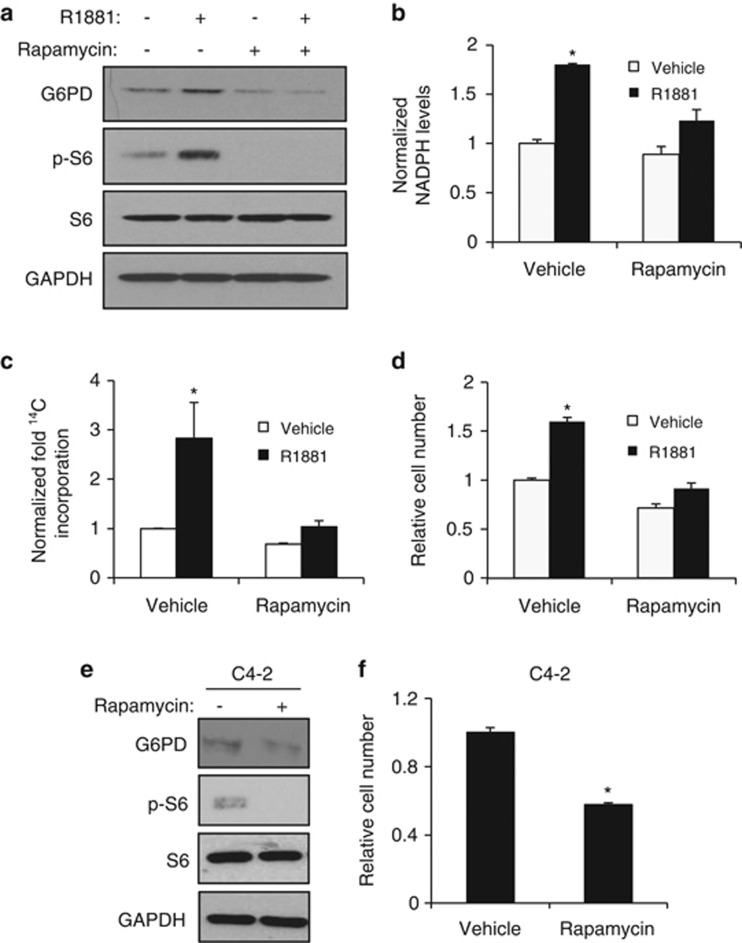
AR regulates G6PD levels via mTOR signaling. (**a**–**d**) LNCaP cells were treated ± 10 nM rapamycin (inhibitor of mTOR) ± 10 nM R1881 for 72 h. Cells were then subjected to immunoblot analysis (**a**), assessed for intracellular levels of NADPH (**b**), flux of ^14^C-labeled glucose into RNA (**c**) or relative cell numbers (**d**) as described above. *, significant (*P*<0.05) changes from vehicle (no R1881). (**e** and **f**) C4-2 cells were treated ± 10 nM rapamycin for 72 h and then subjected to immunoblot analysis (**e**) or assayed for cell growth (**f**). *, significant (*P*<0.05) changes from vehicle (no rapamycin).

Mammalian target of rapamycin (mTOR) is a master regulator of cellular metabolism.^40^ One of the many metabolic pathways mTOR signaling has been shown to regulate is the pentose phosphate pathway, in part by regulating the levels of G6PD.^41^ Previous studies have demonstrated that androgens increase mTOR activity.^42, 43^ Hence, we tested whether AR could be regulating the pentose phosphate pathway through the mTOR-mediated control of G6PD. Accordingly, androgens increased mTOR signaling as assessed by the phosphorylation of S6, an effect that was completely blocked by the mTOR complex 1 (mTORC1) antagonist rapamycin (Figure 5a and Supplementary Figure 4A). These effects subsequently tracked with NADPH levels, nucleic acid synthesis and cell growth (Figures 5b–d and Supplementary Figure 4B). Rapamycin also decreased G6PD levels and cell growth in CRPC cells (Figures 5e and f and Supplementary Figures 4C and D). Taken together, our results suggest G6PD is regulated via the AR–mTOR axis, which is required for pro-growth metabolism.

### G6PD correlates with prostate cancer progression *in vivo*

**Figure 6:**
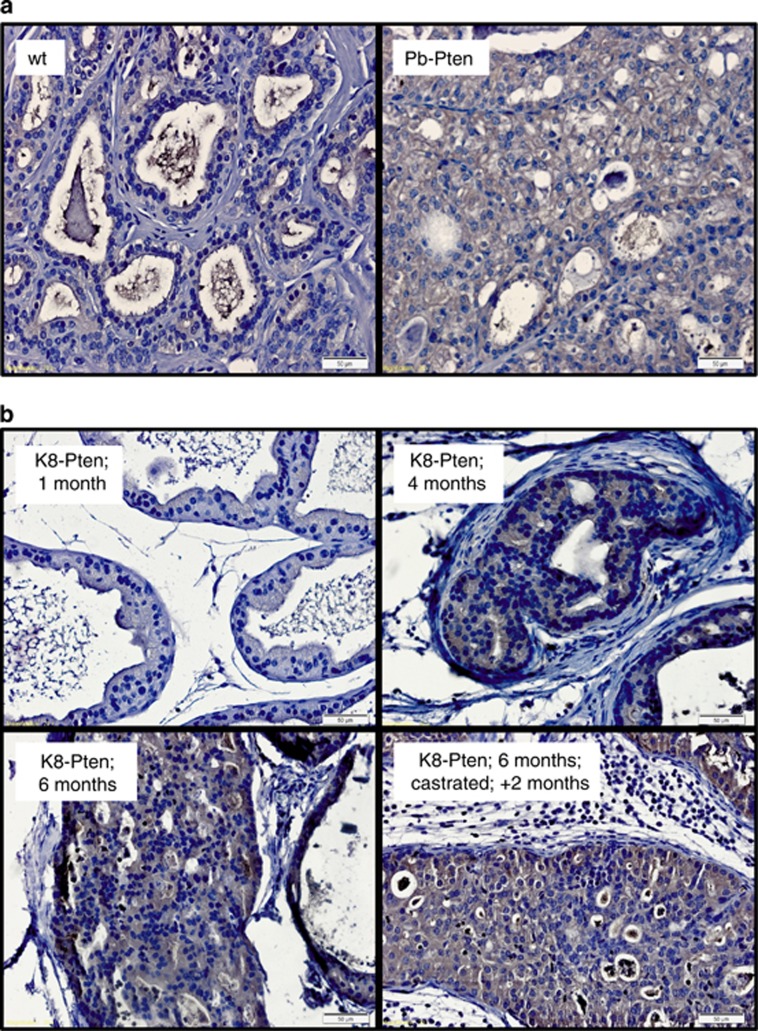
G6PD expression increases during prostate cancer progression *in vivo*. (**a**) Paraffin-embedded prostate tissues from tumors derived from the dorsolateral lobes of 7-week-old Pb-Cre;Pten^f/f^ mice (Pb-Pten) or the dorsolateral lobe of 7-week-old C57BL/6 control mice (wt) were stained for G6PD (brown) and counterstained with hematoxylin (blue). (**b**) Immunohistochemical analysis of G6PD from the dorsolateral lobes of K8-CreER^T2^;Pten^f/f^ inducible transgenic mice (K8-Pten) 1, 4 or 6 months after tamoxifen treatment in intact mice or mice that were castrated 6 months after initial tamoxifen treatment and then killed 2 months after castration. Hematoxylin was again used as a counterstain. White bars=50 μm.

PTEN inactivation is observed in roughly half of all prostate cancers, where its loss-of-function is associated with poor prognosis, aggressive disease phenotype and castration resistance.^44^ PTEN functions as a tumor suppressor by inhibiting phosphoinositide 3-kinase (PI3K) activity and subsequent downstream oncogenic signaling through Akt and mTOR. To study whether G6PD was regulated by mTOR signaling *in vivo*, we performed immunohistochemical analysis of two different autochthonous *in vivo* models of prostate cancer caused by Pten loss-of-function. Prostate cancer caused by the conditional knockout of floxed Pten in the prostate using probasin promoter-driven Cre expression, Pb-Cre;Pten^f/f^ (Pb-Pten), has been previously described.^45^ Following tamoxifen treatment, K8-CreER^T2^; Pten^f/f^ transgenic mice (K8-Pten) develop low-grade prostate intraepithelial neoplasia in 1 month and prostate cancer at 4–6 months.^46, 47^ In both the Pb-Pten (Figure 6a) and K8-Pten (Figure 6b) models, G6PD levels correlated with prostate cancer progression. To determine whether G6PD levels were maintained in CRPC, we castrated K8-Pten mice 6 months after tamoxifen treatment. Following initial tumor regression, 2 months post-castration tumors relapsed^46^ and importantly, continued to express high levels of G6PD (Figure 6b).

## Discussion

Altered glucose metabolism is one of the hallmarks of cancer.^48^ While it was long suspected that this elevated glucose uptake was needed to generate ATP, it is becoming apparent that cancer cells may require sugars for many other oncogenic processes.^3, 4, 5^ For example, rapidly dividing cells require a constant source of building blocks to maintain their heightened rate of biosynthetic reactions. Inherent with this increased metabolism are increased ROS levels, which if left unchecked, could be detrimental to the cell. As such, cancer cells also need to maintain their cellular ROS levels within a window that favors growth. Thus, oncogenic signaling pathways may promote cancer through rerouting sugar metabolism.

An alternative route for glucose metabolism is the pentose phosphate pathway, which can promote both anabolic reactions and redox homeostasis. One of the byproducts of the pentose phosphate pathway is NADPH. NADPH functions as a cofactor and is a reducing agent involved in anabolic reactions as well as the control of ROS levels. The pentose phosphate pathway is the major source of NADPH in the cytoplasm.^17^ In addition to providing the reducing power for many anabolic reactions, NADPH is also needed as an electron donor to replenish the reduced form of glutathione, the major endogenous antioxidant.^49^ Another major product of the pentose phosphate pathway is ribose-5-phosphate. This five-carbon sugar is used in the synthesis of nucleotides and nucleic acids.^50^ Hence, signaling events that promote flux through the pentose phosphate pathway, and particularly the oxidative arm of the pentose phosphate pathway, could engender a cell with clear growth and survival advantages.

Because previous studies implicated elevated G6PD activity in prostate adenocarcinomas compared with normal or benign prostatic hyperplasia tissues,^25, 26^ and G6PD overexpression is observed in several types of cancers,^21, 22, 23^ we focused primarily on this rate-limiting enzyme. While we focused on G6PD and its role in prostate cancer, other groups have demonstrated that additional enzymes involved in the pentose phosphate pathway may be altered in cancer. Indeed, enzymes within both branches of the pentose phosphate pathway, oxidative and non-oxidative, have been found to be upregulated in cancers and contribute to disease progression. In lung cancer, inhibition of 6-phosphogluconate dehydrogenase caused disease regression *in vitro* and *in vivo*.^51^ Further, transaldolase and transketolase, two enzymes of the non-oxidative branch of the pentose phosphate pathway, have been implicated as biomarkers for hepatocellular carcinoma^52^ and breast cancer,^53^ respectively. Whether these enzymes are aberrantly regulated in prostate cancer is unknown.

To understand the mechanism(s) behind G6PD activation, we hypothesized that AR signaling, elevated in the majority of prostate cancers, regulated the levels of this key enzyme. Accordingly, G6PD expression was increased upon androgen treatment in hormone-sensitive prostate cancer cell lines (Figure 4 and Supplementary Figure 3). Conversely, in models of CRPC where basal levels of AR activity drive cell growth, knockdown of AR decreased G6PD expression (Figure 4). As we have shown here (Figure 5), and what is in agreement with what others have previously demonstrated,^42, 43^ AR positively regulates mTOR signaling. Previous studies have shown that mTOR regulates G6PD in HEK231 cells.^41^ mTOR, through the phosphorylation/activation of its downstream target p70S6K, has been shown to activate the sterol-regulatory element binding protein 1 (SREBP1).^54^ This is significant because SREBP1 can regulate *G6PD* expression.^41^ Overexpression of SREBP1 in HEK231 cells or silencing of SREBP1 in mouse embryonic fibroblasts was positively correlated with *G6PD* expression. Additionally, sterol-regulatory elements were found in the promoter region of *G6PD*, indicating a direct regulation of *G6PD* via SREBP1, downstream of mTOR.^41^ Further, SREBP1 is also directly activated by androgens through the increased expression of SREBP cleavage-activating protein,^55, 56, 57^ indicating AR may additionally regulate G6PD through mTOR-independent mechanisms. Interestingly, the levels of G6PD mRNA and protein did not always correlate (Figure 4 and Supplementary Figure 3), suggesting both transcriptional and post-transcriptional regulation by AR. Given mTOR's ability to regulate protein translation,^58, 59^ it is not surprising that mTOR could also post-transcriptionally regulate G6PD levels. Taken together, G6PD, and thus the pentose phosphate pathway, could be regulated by a variety of mechanisms. As such, therapeutically targeting this metabolic pathway by blocking its upstream regulators may prove difficult due to the presence of redundant signaling networks.

Prostate cancers have relatively unique metabolic profiles. Previously, we demonstrated that AR signaling promoted prostate cancer mitochondrial biogenesis and growth through an AMP-activated protein kinase (AMPK) signaling cascade.^38^ In addition, previous studies^42, 43^ as well as our data presented here demonstrate that androgens also increase mTOR signaling. At first glance, these findings appear paradoxical as AMPK and mTOR signaling are known to oppose one another in normal cells.^60^ However, we think the coexistence of both signaling cascades is indicative of the unique metabolism of prostate cancer. By utilizing AMPK and mTOR signaling, cancer cells can enjoy the benefits of both pathways. For example, the activation of AMPK would stimulate increased glucose uptake, which in the presence of active mTOR signaling could then be shuttled into the pentose phosphate pathway to drive anabolic tumor processes. Hence, the loss of a physiological, mutual negative feedback may assist the transition to a pathological phenotype.

A common genetic event that contributes to cancer cell growth and survival is the activation of the PI3K/Akt pathway via PTEN loss-of-function.^61, 62^ In the late stages of the disease, PTEN loss-of-function and/or PI3K/Akt pathway activation is observed in 70% of prostate cancers.^63^ PTEN loss and subsequent PI3K/Akt signaling cause mTOR activation.^64^ mTOR regulates cellular metabolism by controlling glucose uptake, glycolysis, fatty acid metabolism and the pentose phosphate pathway.^41^ mTOR kinase exists in two signaling complexes, mTORC1 and mTORC2. In particular, mTORC1 promotes cell proliferation and anabolic reactions. Several clinical trials are currently underway examining the efficacy of various inhibitors of mTOR signaling.^42, 43, 65, 66^ However, at this time the use of inhibitors of mTOR signaling in patients has been met with limited success, perhaps indicating the presence of redundant mechanisms.^66^ Our results here suggest that metabolism through the pentose phosphate pathway may be a relevant marker for a subset of prostate cancers and that blocking the flux through this pathway may serve as an alternative downstream target to inhibitors of mTOR and AR.

## Materials and methods

### Reagents

6AN and tamoxifen were purchased from Sigma-Aldrich (St Louis, MO, USA). Methyltrienolone (R1881) and ^14^C-labeled glucose (glucose, D-[^14^C(U)]) were purchased from PerkinElmer (Waltham, MA, USA). Rapamycin was purchased from Cell Signaling (Danvers, MA, USA). Anti-GAPDH antibody was from Sigma-Aldrich. Anti-G6PD, anti-pS6 Ser235/236 and anti-S6 antibodies were purchased from Cell Signaling. The NADP/NADPH red fluorescence quantitation assay was from e-ENZYME (Gaithersburg, MD, USA). G6PD siRNAs and Universal siRNA control were purchased from Sigma-Aldrich. Hoechst and MitoSOX were from Life Technologies (Carlsbad, CA, USA).

### Cell culture

LNCaP, VCaP, C4-2, CWR22 and 22Rv1 human prostate cancer cell lines were from ATCC (Manassas, VA, USA) and maintained as previously described.^38^ Androgen-sensitive LAPC4 cells were a gift from Charles L Sawyers) and maintained as previously described.^67^ LNCaP-abl cells were from Zoran Culig and maintained as previously described.^68^ Cells were validated for androgen responsiveness just prior to experiments as previously described.^69^ Prior to all experiments, cells were plated and incubated for 72 h in charcoal-stripped fetal bovine serum to minimize endogenous hormone signaling.

### siRNA transfection

Transient transfection with 100 nM final concentration of siRNAs was performed as previously described.^38^ The sequences of all siRNAs used in this study are listed in Supplementary Table 1.

### RNA isolation, cDNA synthesis and quantitative PCR

RNA isolation, cDNA synthesis and quantitative PCR were performed as described previously using 36B4 as a control.^69^ All primers used in this study are listed in Supplementary Table 1.

### Western blotting

Whole cell lysates were collected and subjected to immunoblot analysis as previously described.^69^ All primary antibody concentrations were 1:1000, except anti-GAPDH that was used at 1:5000. Densitometry was performed using ImageJ software (National Institutes of Health (NIH), Bethesda, MD, USA) and normalized to the indicated control bands.

### Cell proliferation assay

Cell proliferation assays were carried out as previously described,^67^ by measuring the cellular DNA content using a FluoReporter Blue fluorometric double-stranded quantitation kit from Life Technologies following the manufacturer's protocol.

### NADPH measurement assay

Cells were plated in duplicate six-well plates. Following treatments and/or transfection with siRNAs, cells were lysed in NADP/NADPH extraction buffer (Elite fluorimetric NADP/NADPH assay) purchased from e-ENZYME (Gaithersburg, MD, USA). In a 96-well plate, 50 μl extracted lysates were added to 50 μl NADPH recycling buffer. The plate was incubated at room temperature for approximately 2 h and measured at 560/610 Ex/Em. Fluorescence was normalized to the total protein levels of each sample.

### Pentose phosphate pathway flux/^14^C RNA incorporation assay

Cells were plated in six-well plates and allowed to attach for 48 h prior to being transfected with siRNAs or treated ± R1881. After 72 h, medium was changed to starvation media and cells were incubated with 3 μCi D-[^14^C(U)]-glucose, for 24 h at 37 °C, 5% CO_2_. Cells were then lysed and total RNA was extracted. Radioactivity was measured with a scintillation counter. Results were normalized to total RNA concentration.

### ROS measurement assay

Following treatments, cells (∼300–1000 per group) were probed first with 1 μM Hoechst to visualize the nucleus and cell viability, and then with 5 μM MitoSOX to visualize mitochondrial superoxide generation. Following a 1-h incubation, cells were fixed using ice-cold 4% paraformaldehyde at 4 °C overnight. The following day, cells were gently washed three times with phosphate-buffered saline and observed using fluorescence microscopy at × 20 (Olympus XM10, Olympus America, Center Valley, PA, USA). The images were recorded and stored as TIFF files. The pixels of TIFF files were analyzed using ImageJ public domain software (NIH). The total fluorescence intensity was normalized to total cell number for each image.

### Animal studies

Pb-Cre and floxed Pten mice have previously been described.^38^ The K8-CreER^T2^ mice and K8-CreER^T2^;Pten^f/f^ model have also previously been described.^46, 47^ Breeding, crosses, dissociation of prostate tumors and tissue and preparation of paraffin-embedded tissues have previously been described.^70^

### Immunohistochemistry

Paraffin-embedded prostate tissues were deparaffinized in xylene and rehydrated in a gradient concentration of ethanol and remained in H_2_O for 5 min prior to antigen retrieval in sodium citrate buffer. Sections were treated with 3% H_2_O_2_ to eliminate endogenous peroxidase activity and blocked for 30 min with 5% goat serum. Tissues were incubated with anti-G6PD antibody from Abcam (San Francisco, CA, USA) 1:100 overnight at 4 °C. Sections were then incubated with SignalStain Boost detection Reagent (Cell Signaling) for 30 min at room temperature followed by 3,3'-diaminobenzidine staining and hematoxylin counterstain of nuclei.

### Statistical analysis

Student's *t*-tests were used for two sample comparisons. Multiple comparisons were performed by using a one-way analysis of variance, followed by *post hoc* Dunnett's test. Analyses were done using GraphPad Prism, Version 5 (GraphPad Software, La Jolla, CA, USA). All experiments were repeated at least three times unless otherwise noted.
