# Supplementary material for: Regulation of the pentose phosphate pathway by an androgen receptor–mTOR-mediated mechanism and its role in prostate cancer cell growth
Source: Oncogenesis. 2014 May 26;3(5):e103–. doi: 10.1038/oncsis.2014.18 (PMC4035695; doi:10.1038/oncsis.2014.18)

**Supplementary Table 1.** Primers and siRNA sequences used in these studies	
Primer/siRNA	***Sequence***
**qPCR primers**	
36B4	Forward: 5’-GGACATGTTGCTGGCCAATAA-3’<br>Reverse: 5’-GGGCCCGAGACCAGTGTT-3’
G6PD	Forward: 5’-AGAGCTTTTCCAGGGCGAT-3’<br>Reverse: 5’-CACCAGATGGTGGGGTAGAT-3’
**siRNA sequences**	
G6PD #1	5’-CAGAUGACGUCCGUGAUGA-3’
G6PD #2	5’-UCAUCACGGACGUCAUCUG-3’
G6PD #3	5’-CAGAGUGAGCCCUUCUUCA-3’
AR #1	5’-CCCUUUCAAGGGAGGUUACACCAAA-3’
AR #2	5’-UAGAGAGCAAGGCUGCAAAGGAGUC-3’

Supplementary Figure Legends

Supplementary Figure 1. G6PD is required for maximal prostate cancer cell proliferation. (**a**) hormone-sensitive LAPC4 cells were treated ± 100 nM 6-aminonicotinamide (6AN) ± the synthetic androgen R1881 for 7 days. Relative cell numbers were quantified after cell lysis by a fluorescent DNA-binding dye. *, significant (*P*<0.05) changes from vehicle (no R1881). (**b**) CRPC 22Rv1 cells were treated ± 100 nM 6AN for 7 days. Relative cell numbers were quantified as in **a**. *, significant (*P*<0.05) changes from vehicle (no 6AN). (**c** and **d**) CWR22 (**c**) and 22Rv1 (**d**) cells were transfected with siRNAs targeting scramble control (siCtrl) or G6PD (no. 1-3). 72 hours post-transfection, cells were harvested and subjected to immunoblot analysis using GAPDH as a loading control. (**e** and **f**) CWR22 (**e**) and 22Rv1 (**f**) cells were transfected with siRNAs as described in **c** and **d**. Seven days later, relative cell numbers were quantified as in **a**. Representative results are expressed as mean relative cell number + SE. *, significant (*P*<0.05) changes from control siRNA.

Supplementary Figure 2. Androgens increase NADPH levels in a dose-dependent manner. LNCaP cells were treated with increasing R1881 concentrations (0, 0.1, 1 and 10 nM) for 72 hours. Cells were lysed and NADPH was measured and normalized to total protein concentration.

Supplementary Figure 3. AR regulates G6PD expression and prostate cancer cell growth. (**a**) VCaP cells were treated with increasing concentrations of R1881 (0, 0.01, 0.1, 1 and 10 nM) for 24 or 72 hours or treated ± 10 nM R1881 for 24, 48 or 72 hours. Protein lysates were subjected to immunoblot analysis using GAPDH as a loading control. (**b**) Comparison of G6PD protein levels in parental hormone-sensitive CWR22 and LNCaP cell lines to their CRPC-derivative 22Rv1 and LNCaP-abl cell lines, respectively. (**c**) LNCaP, LAPC4 and VCaP cells were treated with ± 10 nM R1881 for 24 or 72 hours. Total RNA was

extracted and subjected to qPCR analysis to detect G6PD mRNA levels. Data were normalized to 36B4 levels. *, significant (*P*<0.05) changes from vehicle.

Supplementary Figure 4. AR regulates G6PD levels and prostate cancer cell growth through mTOR signaling. (**a** and **b**) LAPC4 cells were treated ± 10 nM rapamycin ± 10 nM R1881 for 72 hours. Cells were then subjected to immunoblot analysis (**a**) or subjected to the ^{14}C RNA incorporation assay (**b**) as described in Figure 3. (**c** and **d**) 22Rv1 cells were treated ± 10 nM rapamycin for 72 hours and then subjected to immunoblot analysis (**c**) or assayed for cell growth (**d**). *, significant (*P*<0.05) changes from vehicle.

Supplemental Figure 1

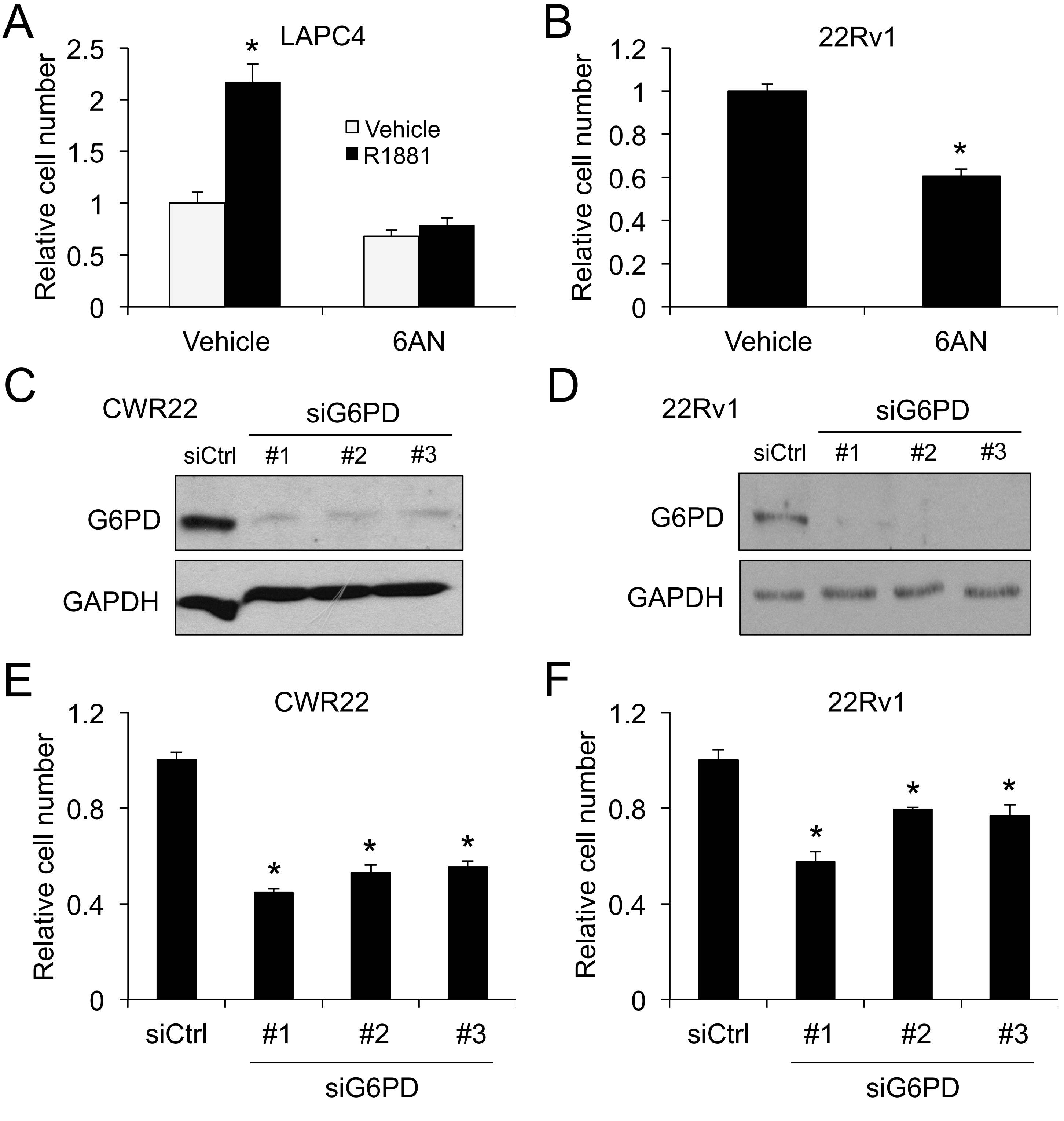

Supplemental Figure 2

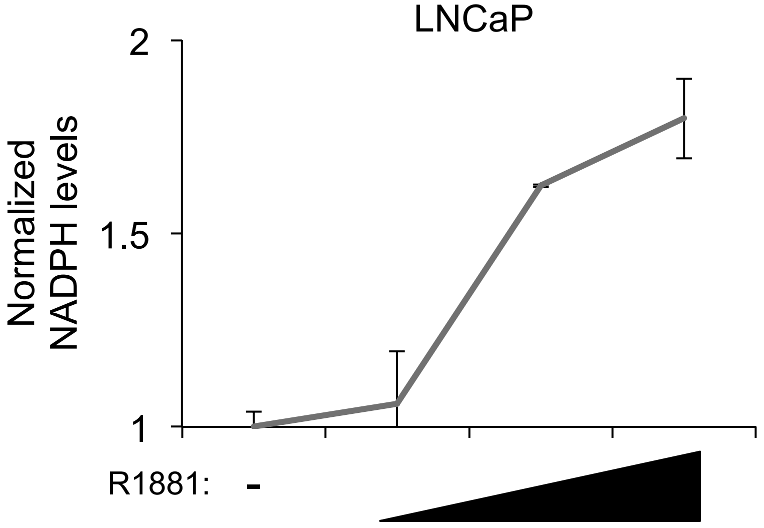

Supplemental Figure 3

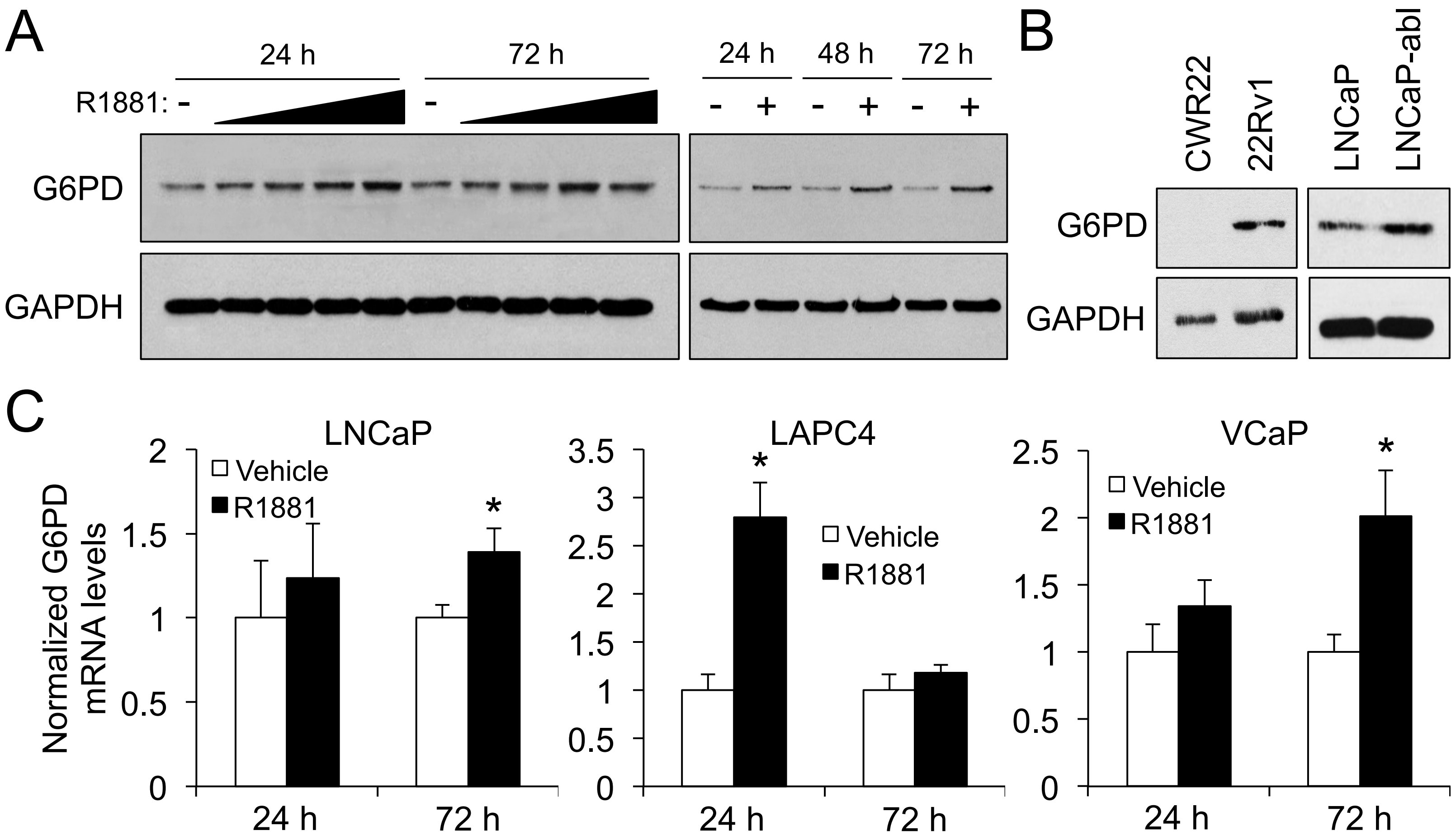

Supplemental Figure 4

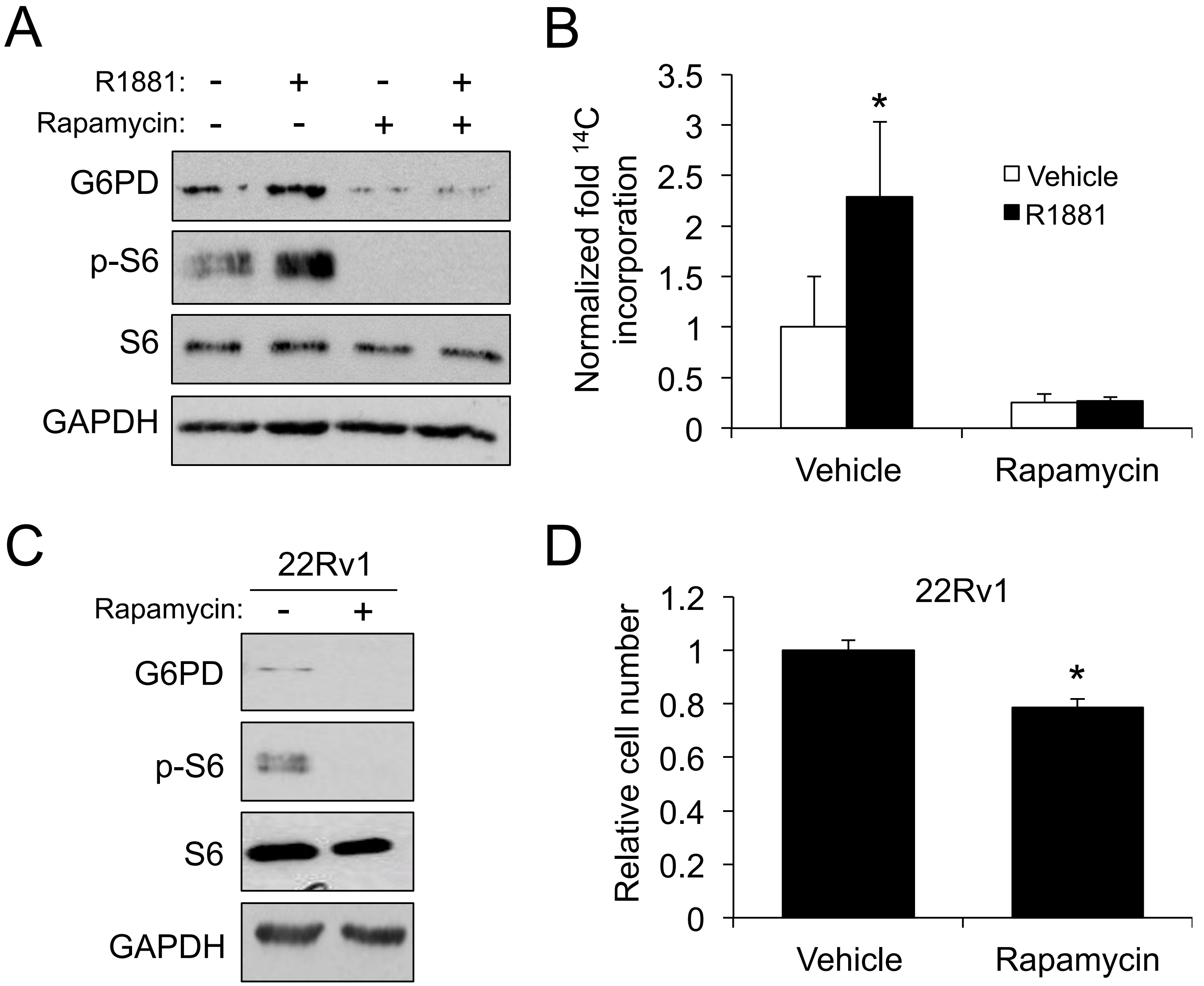

Supplement: Supplementary Information [file oncsis201418x1.pdf]
